# Longitudinal Variations in Retinal and Neuronal Degeneration in Cognitively Normal Older Adults using Peripapillary Optical Coherence Tomography Angiography

**DOI:** 10.1002/alz.086538

**Published:** 2025-01-09

**Authors:** Joshua Woo, Sharon Fekrat, Suzanna Joseph, Cason B Robbins, Alice Haystead, Sandra Stinnett, Dilraj S Grewal

**Affiliations:** ^1^ Duke University School of Medicine, Durham, NC USA; ^2^ Duke Eye Center, Durham, NC USA; ^3^ Duke University, Durham, NC USA

## Abstract

**Background:**

Despite an aging population, it remains challenging to reliably differentiate between loss of cognitive function associated with normal aging and cognitive decline associated with pathologic processes. With growing interest in using retinal and optic nerve biomarkers to diagnose neurodegenerative diseases, characterization of the velocity of normal retinal age‐related changes will further our understanding. We evaluated longitudinal microvascular changes in cognitively normal older adults using optical coherence tomography (OCT) and OCT angiography (OCTA).

**Methods:**

Participants over 50 years old without history of neurodegenerative disease or cognitive impairment were enrolled. Exclusion criteria included retinal/optic nerve pathology, glaucoma, and diabetes, among others. The Zeiss Cirrus HD‐OCT 5000 with AngioPlex was used to obtain OCT and OCTA images at baseline and two years later. OCTA parameters measured were optic nerve head‐centered capillary perfusion density (CPD) and capillary flux index (CFI). OCT was used to measure retinal nerve fiber layer (RNFL) thickness. Generalized estimating equations were used to account for measurements obtained from two eyes of the same subject.

**Results:**

189 eyes of 111 cognitively normal older adults were analyzed (average age at baseline, 69.3 ± 5.8; average Mini‐Mental State Examination score, 29.54 ± 0.71; 86 women [77.5%]). Average follow up was 2.1 ± 0.5 years. After controlling for sex, CPD (p<.001), CFI (p<.001), and RNFL thickness (p=0.005) decreased with increasing age at both cross‐sectional time points. Over the follow‐up period, rate of change in CPD, CFI, and RNFL thickness was not significantly correlated with age; however, rate of change in CPD (p=.038) and RNFL thickness (p=0.008) was slower in females compared to males. Average CPD was higher among females at both time points compared to males (p=.002), with no significant differences in CFI and RNFL thickness.

**Conclusions:**

In cognitively normal adults, there is a significant reduction in peripapillary CPD, CFI, and RNFL thickness associated with aging beyond 50 years old. Females had higher CPD values with slower rates of change in CPD and RNFL thickness. With prior studies documenting microvascular alterations associated with Alzheimer’s disease, these values provide benchmarks of normal aging and help detect accelerated rates of decline in neurodegenerative diseases.

